# Mortality Statistics, Chicago

**Published:** 1875-05

**Authors:** 


BOARD OF HEALTH, OFFICE SANITARY SUP'T,
CHICAGO, April 7, 1875.

*Dr. W. Hay, Editor Journal:*

DEAR SIR—I transmit herewith the report for March, also some figures rom our annual report, (the fiscal year ending April 1st). You will see that the decrease is 1,532, compared with preceding year, and 2,131, com pared with 1872. The decrease from small-pox was 427 deaths, and the decrease in the number of cases was 1,462; compared with 1872, the decrease was 1,939 cases. The prevalent diseases of this climate show a marked decrease. Taking everything into consideration, the death rate for the year has been very low. Respectfully,

BEN. C. MILLER, *San. Supt.*

| | | |
|---|---|---|
| The total number of deaths for 1874 | | 8,025 |
| For the preceding year | | 9,557 |
| Deaths from small-pox | 90 | |
| Deaths from small-pox preceding year | 517 | |
| Decrease | 427 | |
| Compared with 1872, the decrease was | 565 | |
| The decrease in the number of cases compared with preceding year | | 1,462 |
| Decrease compared with 1872 | | 1,939 |
| Decrease by— | | |
| Consumption | | 10 |
| Convulsions | | 171 |
| Pneumonia | | 31 |
| Diseases of the brain | | 231 |
| Diarrhœa | | 36 |
| Diphtheria | | 14 |
| By cholera infantum there were more deaths | | 66 |

During the month of March the report of Dr. Reid, health officer, shows:—

| | |
|---|---|
| Notices served | 1,273 |
| Nuisances abated | 759 |
| Articles condemned as unfit for food— | |
| Quarters of Beef | 288 |
| Carcasses of mutton | 9 |
| Calves | 134 |
| Hogs | 21 |
| Fresh meat (lbs.) | 1,532 |
| Corned beef (lbs.) | 3,460 |
| Hams | 311 |
| Shoulders | 285 |
| Ducks | 12 |
| Dead animals removed during the month | 906 |
| Suits brought for violating health ordinance | — |

***Chicago Mortality Report for March, 1875.*** Reported by Dr. BEN. C. MILLER, Sanitary Superintendent.

| | | | |
|---|---|---|---|
| Accident, by fall | 3 | Aorta, atheroma of | 1 |
| " by drowning | 1 | Ascites | 2 |
| " by railroad | 3 | Atelectasis pulmonum | 1 |
| Abscesses | 1 | Bladder, inflammation of | 1 |
| Anus, imperforate | 1 | Bowels, congestion of | 2 |
| Apoplexy | 7 | " hæmorrhage of | 1 |
| Asphyxia | 1 | Brain, anæmia of | 1 |

| | |
|---|---|
| Brain, abscess of | 2 |
| " compression of | 1 |
| " congestion of | 6 |
| " disease of | 1 |
| " inflammation of | 5 |
| " softening of | 1 |
| Bronchitis | 14 |
| " capillary | 3 |
| Cancer of face | 1 |
| " of liver | 4 |
| " of stomach | 3 |
| " of uterus | 1 |
| Cholera infantum | 1 |
| Colon, stricture of | 1 |
| Consumption | 71 |
| Convulsions | 66 |
| Croup | 6 |
| " membranous | 1 |
| Cyanosis | 2 |
| Debility, general | 4 |
| Diarrhœa | 2 |
| " chronic | 1 |
| Diphtheria | 9 |
| Dropsy, general | 8 |
| Dysentery | 2 |
| Embolism | 1 |
| Endo-carditis | 1 |
| Enteritis | 10 |
| Entero-colitis | 1 |
| Exposure | 1 |
| Erysipelas | 4 |
| Fever, congestive | 1 |
| " puerperal | 8 |
| " remittent | 1 |
| " scarlet | 12 |
| " typhoid | 10 |
| Gangrene of leg | 1 |
| Gastritis | 3 |
| Hæmorrhage | 1 |
| Heart, disease of | 8 |
| " fatty degeneration of | 4 |
| " hypertrophy of | 1 |
| " mitral valves, disease of | 1 |
| " rheumatism of | 3 |
| " paralysis of | 2 |
| " valvular disease of | 4 |
| Hemiplegia | 1 |
| Hepatitis | 1 |
| Hernia, strangulated | 2 |
| " congenital | 1 |
| Hydrocephalus | 4 |
| Inanition | 9 |
| Jaundice | 2 |
| Kidneys, disease of | 1 |
| " Bright's disease of | 5 |
| Liver, cirrhosis of | 1 |
| " inflammation of | 1 |
| Lungs, congestion of | 7 |
| " hæmorrhage of | 1 |
| Manslaughter | 2 |
| Mania, puerperal | 1 |
| Measles | 18 |
| " typhoid | 1 |
| Meningitis | 8 |
| " cerebro-spinal | 9 |
| " tubercular | 4 |
| Metritis | 1 |
| Œdema, pulmonum | 1 |
| Old age | 13 |
| Paralysis | 3 |
| Pericarditis | 1 |
| Peritonitis | 5 |
| " puerperal | 3 |
| Pneumonia | 63 |
| " typhoid | 5 |
| Pleurisy | 2 |
| Poisoned | 3 |
| Pyæmia | 3 |
| Rheumatism | 3 |
| " inflammatory | 1 |
| Scrofula | 1 |
| Suicide, by poison | 1 |
| " by hanging | 2 |
| " by morphine | 3 |
| Septicæmia | 2 |
| Small-Pox | 3 |
| Spine, disease of | 4 |
| " paralysis of | 1 |
| Stomach, softening of | 1 |
| Syphilis | 2 |
| Tabes mesenterica | 4 |
| Teething | 5 |
| Tetanus | 1 |
| Trismus | 2 |
| Thrush | 1 |
| Urine, retention of | 1 |
| Uræmia | 1 |
| Uterus, hæmorrhage of | 1 |
| Vitality, deficient | 2 |
| Whooping cough | 4 |
| Unknown | 1 |
| Total | 549 |

Premature births, 7; Still births, 68. Total, 75.

COMPARISON.

Deaths in March, 1875, 549; in February, 1875, 460. Increase, 89. Deaths in March, 1874, 508. Increase, 41.

AGES.

| | | | |
|---|---|---|---|
| Under one year | 163 | Thirty years to forty | 67 |
| One year to two | 49 | Forty " " fifty | 40 |
| Two years to three | 28 | Fifty " " sixty | 31 |
| Three " " four | 22 | Sixty " " seventy | 30 |
| Four " " five | 12 | Seventy " " eighty | 21 |
| Five " " ten | 13 | Eighty " " ninety | 4 |
| Ten " " twenty | 20 | Ninety " " one hundred | — |
| Twenty " " thirty | 48 | One hundred and upwards | 1 |
| | | Total | 549 |

| | | | | | |
|---|---|---|---|---|---|
| White | 540 | Males | 312 | Married | 193 |
| Colored | 9 | Females | 237 | Single | 356 |
| Total | 549 | Total | 549 | Total | 549 |

NATIVITIES.

| | | | | | |
|---|---|---|---|---|---|
| Austria | 1 | England | 13 | Russia | 1 |
| Belgium | 1 | France | 2 | Scotland | 6 |
| Bohemia | 6 | Germany | 66 | Sweden | 8 |
| Canada | 3 | Holland | 2 | Switzerland | 1 |
| Native—Chicago | 80 | Ireland | 61 | Unknown | 5 |
| Foreign, " | 176 | Italy | 2 | | |
| U. States, other parts | 100 | Norway | 10 | Total | 549 |
| Denmark | 3 | Poland | 3 | | |

Deaths daily, 17⅗. Mean thermometer, 31.5°. Rain fall, 1.37 inches.

MORTALITY BY WARDS.

| Wards. | No. Deaths. | Pop. in 1874. | Percentage. |
|---|---|---|---|
| 1 | 6 | 5,725 | one death in 954 |
| 2 | 4 | 4,830 | " " 1,207 |
| 3 | 20 | 14,861 | " " 743 |
| 4 | 18 | 15,361 | " " 853 |
| 5 | 30 | 20,078 | " " 669 |
| 6 | 54 | 35,916 | " " 665 |
| 7 | 42 | 31,722 | " " 755 |
| 8 | 45 | 29,143 | " " 648 |
| 9 | 36 | 31,654 | " " 879 |
| 10 | 16 | 17,385 | " " 1,087 |
| 11 | 18 | 14,022 | " " 779 |
| 12 | 21 | 16,792 | " " 799 |
| 13 | 10 | 17,892 | " " 1,789 |
| 14 | 23 | 16,720 | " " 727 |
| 15 | 56 | 45,545 | " " 813 |
| 16 | 25 | 21,922 | " " 877 |
| 17 | 36 | 20,777 | " " 577 |
| 18 | 24 | 21,392 | " " 891 |
| 19 | 5 | 4,677 | " " 935 |
| 20 | 8 | 8,995 | " " 1,124 |
| | 497 | Ratio of deaths to population in 1874, one death in 720½. | |

| | | | |
|---|---|---|---|
| No. deaths in Wards | 497 | Manslaughter | 2 |
| Accidents | 7 | Protestant Orphan Asylum | 3 |
| County Hospital | 6 | Poisoned | 3 |
| Foundlings' Home | 14 | St. Joseph's Hospital | 3 |
| Home for Friendless | 2 | Suicides | 6 |
| Hospital Alexian Bros. | 1 | | |
| Mercy Hospital | 5 | Total | 549 |

